# Comparison of different topical anesthetic methods for intravitreal injections: a randomized crossover study

**DOI:** 10.1186/s40942-025-00649-6

**Published:** 2025-03-26

**Authors:** Jeffrey Man Yeung Lo, Veronica Yui Yan Li, Rachel Ka Ying Cheung, Shing Chuen Chow, Kendrick Co Shih, Nicholas Siu Kay Fung, Wai-Ching Lam

**Affiliations:** 1 Department of Ophthalmology, Grantham Hospital, Hong Kong SAR, China; 2 Department of Ophthalmology, School of Clinical Medicine, The University of Hong Kong, Hong Kong SAR, China; 3 Hong Kong Sanatorium and Hospital, Happy Valley, Hong Kong SAR, China; 4 Department of Ophthalmology & Visual Sciences, University of British Columbia, British Columbia, Canada

**Keywords:** Intravitreal injection, Anesthesia, Pain management

## Abstract

**Background:**

To evaluate the potential adjunctive effect of pledget anesthetic to topical proparacaine applied in a droplet form in patients undergoing intravitreal injections (IVI).

**Method:**

This is a single-centre, prospective, randomized, double-blinded crossover study. 60 patients were included. Patients receiving IVI were given topical 0.5% proparacaine drops then randomized in a 1:1 ratio to receive 0.5% proparacaine soaked pledget or normal saline soaked pledget as placebo. The patients would later be crossed over to receive the alternative intervention. Pain was assessed with a visual analog scale (VAS) and questionnaire immediately afterwards, 10-minutes and 20-minutes after injection.

**Result:**

Pain intensity as assessed on the visual analogue scale was lower for the placebo group compared to the pledget group immediately (2.51 cm vs. 2.8 cm), 10-minutes (1.81 cm vs. 2.13 cm) and 20-minutes (1.23 cm vs. 1.65 cm) after injection, however this was not statistically significant (*p* = 0.48, *p* = 0.43, *p* = 0.24 respectively). However, in a subgroup of treatment naïve patients, the addition of pledget anesthesia may lower pain and make IVI more tolerable.

**Conclusion:**

Additional pledget soaked with proparacaine does not enhance anesthesia compared to solely using topical proparacaine for IVI, except in a subset of treatment naïve patients.

**Supplementary Information:**

The online version contains supplementary material available at 10.1186/s40942-025-00649-6.

## Background

Intravitreal injection (IVI) is the standard of delivering therapeutic agents to the back of the eye. It has become the mainstay of treatment for various conditions including age-related macular degeneration, diabetic macular edema, macular edema due to retinal venous occlusion and other exudative maculopathies. In the past decade, it has become the most commonly performed ophthalmic procedure [1]. It has increased nearly 11-fold from 2009 to 2019, with 44,924 injections done in 2019 at Moorfields Eye Hospital alone and an estimated 6 million injections in the USA in 2016 [2]. With an ageing population and the advent of newer therapeutic agents, that number is expected to continue to increase.

The injection procedure can be quite distressing and unpleasant [3]. Patients have expressed anxiety associated with the needle puncture as well as discomfort with the disinfection process, and lid speculum [4]. Different types and forms of anesthesia have been used during IVI to help alleviate the discomfort associated with injections. These include topical eye drops, topical gels, pledgets, and subconjunctival injections. Commonly used drugs in topical anesthesia include proparacaine hydrochloride (HCL), tetracaine HCL, and lignocaine. They exert their action by inhibiting sodium ion influx through voltage-gated sodium channels, which prevents the initiation of neural impulses [5]. Proparacaine has become a particularly popular choice due to its rapid onset of action and lack of discomfort during instillation [5]. Previous studies of different agents did not show any difference in overall pain scores and a wide range of agents and techniques are still commonly used [6–8]. A less popular choice however are topical gels due to potentially increased microbial survivability and thus higher rate of endophthalmitis [9, 10]. 

With the number of IVIs increasing, the burden of injections will continue to rise. There is currently no gold-standard in anesthetic method in order to minimize discomfort. We aim to compare the enhancing anesthetic effect of pledget form of proparacaine HCL to that of just the droplet form alone. We hypothesize that pledget of anesthesia would enhance pain control via two mechanisms. Firstly, pledget of anesthesia provides a localized and concentrated area of conjunctival surface contact compared to droplet form, which spreads more diffusely across the surface of the eye. And secondly, the application of a pledget may provide transient pressure anesthesia. Pressure anesthesia involves application of external pressure, thereby suppressing nerve conduction and inhibiting pain sensation [11]. 

## Methods

This is a single-centre, prospective, randomized, double-blinded, placebo controlled, crossover and interventional study. The study was carried out at Grantham Hospital, Hong Kong from October 2021 to June 2022. The study received approval by the governing research and ethics committee (Institutional Review Board of the University of Hong Kong/ Hospital Authority Hong Kong West Cluster (“HKU/HA HKWC IRB”)), (UW 21–526) and was conducted in accordance with the principles of the declaration of Helsinki. The study questionnaire was clearly explained to all participants and informed consent was obtained prior to the study.

**Fig. 1:**
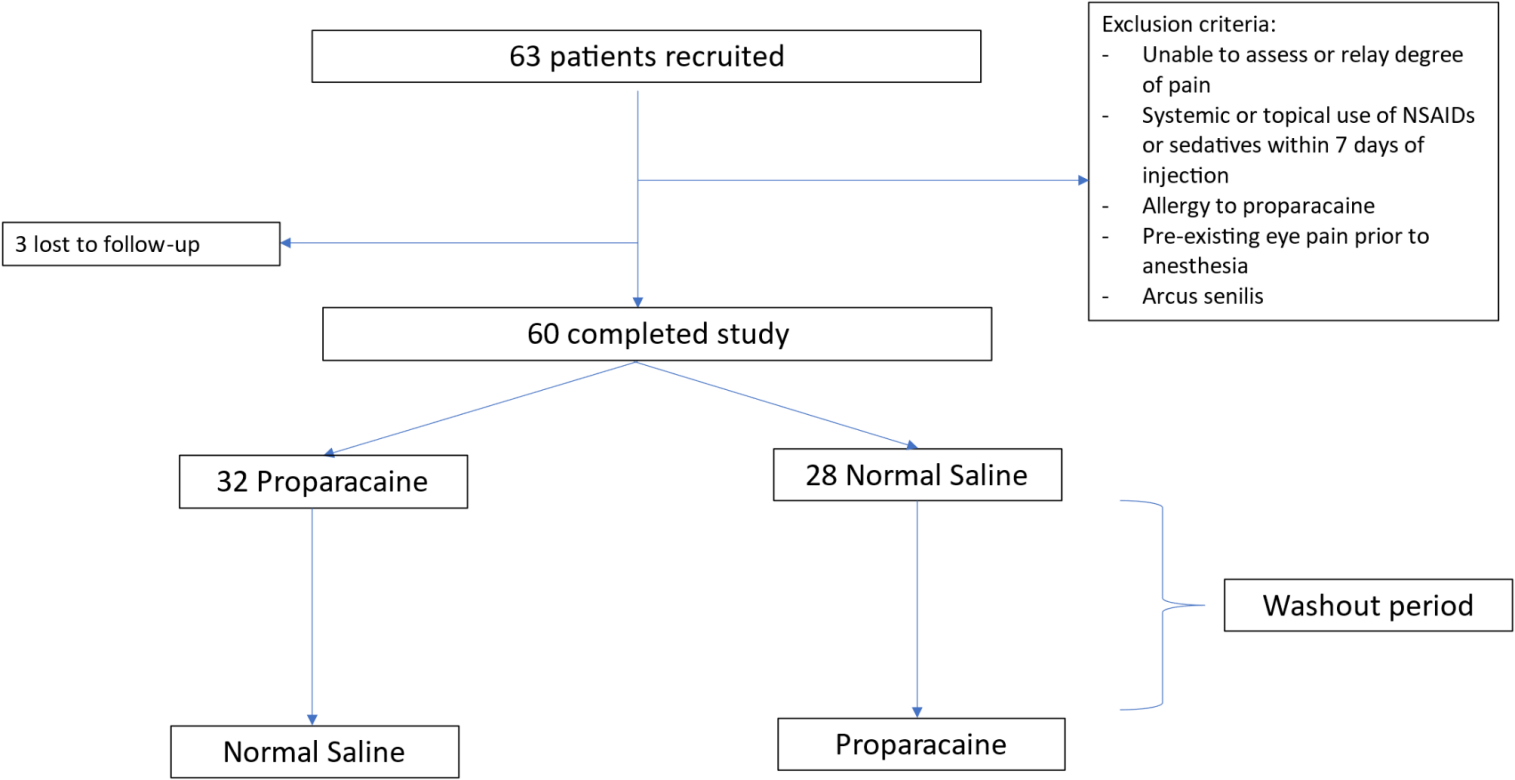
Study profile

Patients who meet the inclusion and exclusion criteria (see Table 1) were randomly assigned in a 1:1 ratio using block randomization, to receive either 0.5% proparacaine soaked pledget, or placebo with pledget soaked in normal saline (NS) after topical 0.5% proparacaine was applied in both arms. (Fig. 1).

At the subsequent visit for IVI, the patients were crossed over to receive the alternative intervention. Each IVI was separated by a washout period ranging from 4 to 16 weeks. A period of at least 4 weeks was deemed to be sufficiently long to rule out any carryover effect.

For the study group, a pledget in the form of a cotton tipped swab, was soaked in 0.5% proparacaine and applied to the injection site for 1 min. The placebo group received a cotton tipped swab soaked in NS.

Prior to the injection, patients were asked if they have been suffering from any ocular pain, which could act as a confounding factor. These patients were excluded from the study.

For each IVI, the surgical nurse prepared a pledget soaked with either NS or 0.5% proparacaine, according to the randomized instructions in a sealed opaque envelope. Thus, the doctor applying the pledget and administering the intravitreal injection was blinded from the assignment.

### Procedure of intravitreal injection

All pledget administration and IVIs were performed in the operating theater with the same technique and under aseptic conditions. The injections were administered by one of five resident doctors (C.L., J.L., J.P., R.C., L.W.Y.) with the same standardized technique. Prior to injections, all patients received one drop of topical proparacaine HCI 0.5% every five minutes for three times prior to injection. Patients would then receive a pledget of either NS or proparacaine for 1-minute, placed in the superotemporal quadrant directed at the site of injection. Topical 5% povidone iodine was then used to disinfect the ocular surface and 10% povidone iodine was used to disinfect the eyelashes and eyelids. A sterile drape was placed over the eye and an eyelid speculum was inserted. Intravitreal injection of 0.05 ml was performed at the superotemporal quadrant 3.5–4 mm posterior to the limbus, using a 30-gauge needle in a straight in straight out manner.

**Table 1:** Inclusion and exclusion criteria for patient recruitment

Inclusion Criteria	
1. Patient aged above 18 2. Patients requiring intravitreal injection and scheduled for another intravitreal injection in the subsequent 3 months	
**Exclusion Criteria**	
1. Individuals with impaired mental capacity who is unable to assess and relay the degree of pain experienced to the physician 2. Individuals with previous eye surgery apart from cataract extraction surgery, and ocular comorbidities that may alter ocular pain or cause ocular neuropathy including herpetic eye disease, uncontrolled glaucoma, uveitis, active conjunctivitis, keratitis, arcus senilis and bullous keratopathy 3. Individuals with previously known allergy to proparacaine 4. Individuals with any systemic or topical use of NSAIDs or sedative medications within 7 days from the current visit (including the day of IVI) 5. Individuals with eye pain prior to the anesthesia administration, to minimize confounding	

### Pain evaluation

Following the IVI, the subjects recorded their pain levels on a VAS (appendix 1) immediately, 10 min and 20 min post injection. These correspond to questions 2, 3, and 4 of the questionnaire respectively.

The primary outcome was the pain intensity as reported on the VAS. In addition to pain intensity, we also recorded the sequence of pledget intervention, age, sex, number of prior injections and indication for treatment.

### Statistical analysis

A power analysis was performed and the minimum sample size required was calculated as at least 60 to reach a significance level of < 0.05. The sample size calculation was based on similar previous crossover studies [12, 13]. 

All statistical analysis was done using Excel (Microsoft Corp., Redmond, WA, USA) and SPSS version 23.0 (IBM Corp., Chicago, USA). All data sets were analysed using a two-sample t-test for equal variance.

## Results

63 patients were enrolled in the study and 60 completed the study. 2 dropped out due to financial restraints of IVI and 1 dropped out from discomfort associated with the first injection. There were 43 males and 17 females. The mean age of the patients was 72 ± 9.2. The patients had undergone a mean of 8.8 IVIs, and there were 11 treatment naive patients (Table 2).

**Table 2:** Patient demographics of the 60 enrolled patients

Characteristics	Number of patients
Age (mean ± SD):	72 ± 9.2
Sex	
Male	43
Female	17
Indication	
AMD	39
DME	12
CRVO	2
BRVO	6
Macular telangiectasia	1
Average number of previous injections	8.8 ± 11.1
Number of treatment naive patients	11
Mean age	68.5 ± 8.1
Sex	Female 2, Male 9
Indication:	
AMD	6
RVO	3
DME	1
Macular telangiectasia	1

**Fig. 2:**
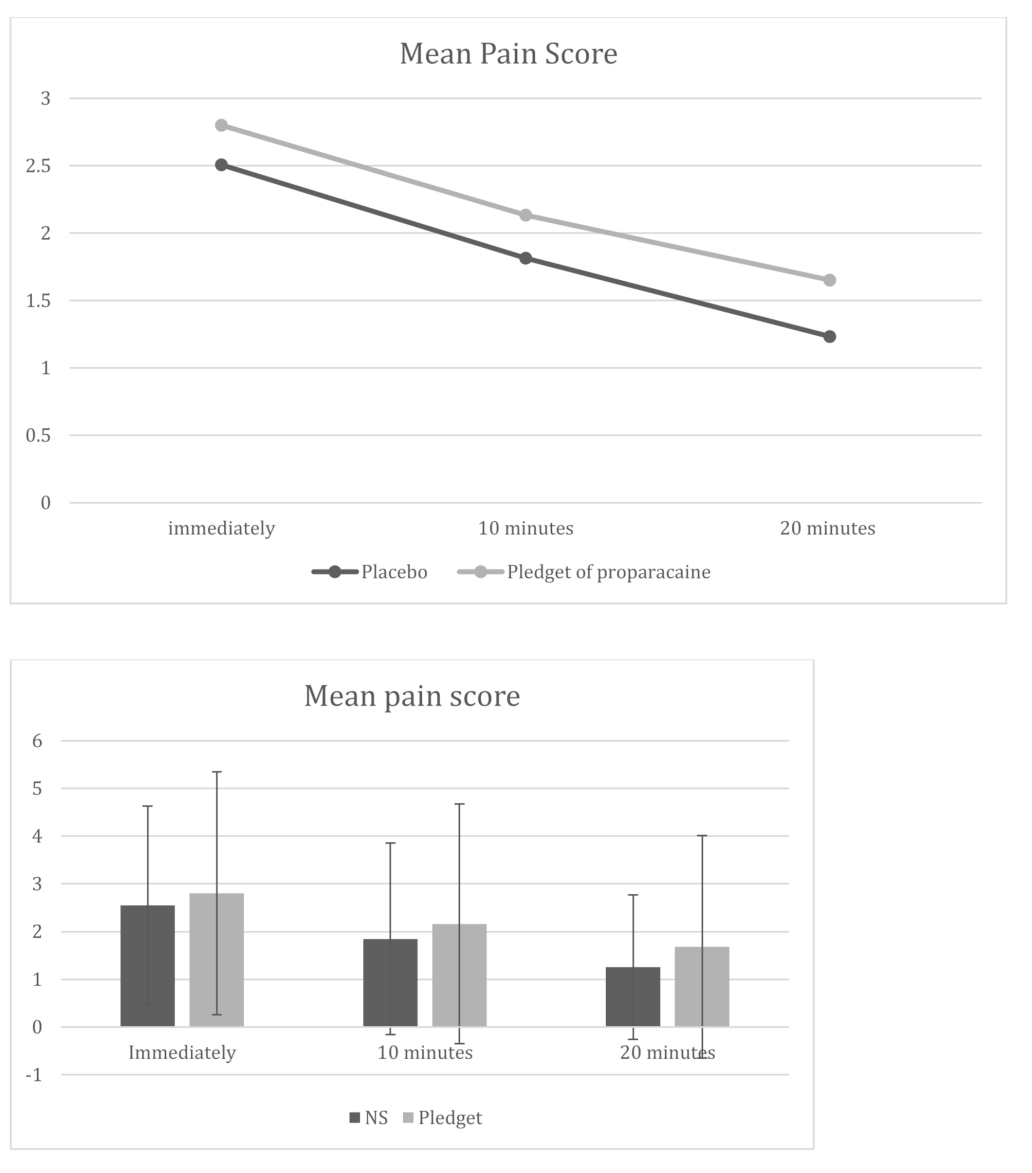
Overall trend of pain scores for the two different topical anesthetic methods illustrated with line and bar graphs

The mean pain scores immediately, 10-minutes and 20-minutes post injection were all lower for the topical proparacaine group compared to the pledget with proparacaine group, however the difference was not statistically significant. (Table 3; Fig. 2).

**Table 3:** Comparison of mean pain scores on visual analogue scale

Mean pain score on visual analogue scale ± SD (range)	Placebo (Pledget of NS)	Test subjects (Pledget 0.5% proparacaine)	*P* value
Immediate	2.51 ± 2.08 (0-8.5)	2.80 ± 2.54 (0-9.8)	0.48
10 min after	1.81 ± 2.00 (0-9.2)	2.13 ± 2.51 (0–9)	0.43
20 min after	1.23 ± 1.51 (0–6)	1.65 ± 2.34 (0-8.8)	0.24

Some patients experience discomfort to some degree regarding the use of the cotton swab. According to the results of Q5a, 26 patients responded that they experienced discomfort or pain associated with the use of the cotton swab for both proparacaine and placebo groups, amounting to 42.6% of all participants (*n* = 60). According to Q6a, after the procedure, 8 patients responded they had discomfort with blinking in the placebo group and 10 patients in the proparacaine group.

There were 11 treatment naïve patients in this study. They had a mean age of 68.5 ± 8.1 and there were 9 males and 2 females. Six of the 11 naive cases received pledget with NS first and 5 received pledget with proparacaine first. There was no significant baseline difference between the treatment naïve group and those with prior injection experience. For treatment naïve patients, the pain score was higher for the topical group 2.97 compared to the pledget group 2.12 immediately after injection, although this was not statistically significant *P* = 0.31. (Table 4). From the treatment naïve patients, 6 were initially assigned to NS and 5 initially assigned to pledget.

**Table 4:** Pain score of treatment Naïve patients

Mean pain score on visual analogue scale (*n* = 11)	Placebo (Pledget of NS)	Test subjects (Pledget 0.5% proparacaine)	*P* value
Immediate	2.97	2.12	0.31
10 min after	2.81	1.41	0.10
20 min after	2.25	0.85	0.08

Further subgroup analysis was done for AMD and DME patients. For AMD patients, the pain score was higher for NS group (2.77) compared to pledget group (2.63) immediately after injection (*P* = 0.40). For DME patients, the pain score was higher for the pledget group (3.04) compared to NS group (2.07) immediately after injection (*P* = 0.18).

## Discussion

Over the past decade, anti-VEGF has revolutionized the treatment of many posterior segment disorders, making it one of the fastest growing procedures done clinically. With the high volume of injections being performed, the pain and anxiety associated with injections may pose a burden to our patients. Various analgesic methods are currently used to help relieve pain, however there is no current gold-standard. Our study aimed to compare a pledget form to a droplet form of topical anesthesia and to determine whether pledget would further enhance anesthesia.

Our results did not show any additional analgesic benefit of pledget anesthesia compared to topical anesthetic drops and that pledget does not have an adjunctive effect to the topical anesthesia. The pledget form of anesthesia did not contribute to additional levels of pain control on VAS when compared with topical droplets alone. Having been used in past studies to evaluate pain after ophthalmic procedures including phacoemulsification, photorefractive surgeries and IVIs, VAS was chosen as our primary outcome measure [14–16]. It was chosen because pain is a highly subjective experience and VAS provides a more nuanced grading system with less discrete levels. However, for such a subjective perception such as pain, another outcome measure such as a questionnaire may have provided additional information.

Currently, no one method of topical anesthesia for intravitreal injections has been proven optimal. This is compatible with previous studies, which have also shown that no single anesthetic or delivery mechanism provides superior anesthesia to others in a statistically significant way [17]. A systematic review by Han et al. looked at pain management for IVIs and efficacy of different anesthesia, including eyedrops, gels, pledgets and subconjunctival injections. 12 articles were included and 3 specifically looked at pledgets. Pledget remains a popular choice of anesthesia as a survey by Xing et al. in 2014 showed that 23% of Canadian specialists routinely used pledget soaked with tetracaine or proparacaine [8]. All 3 studies conducted by Yau, Blaha and David et al. administered a drop of topical anesthetic prior to pledget administration and found similar pain scores compared to drops alone [7, 18, 19]. Topical anesthetic drops were the most popular in the survey conducted by Xing et al., which revealed that 90% of Canadian retina specialists used eyedrops for IVI [8]. 1 study by Andrade et al. found a statistically significant decrease in pain score with subconjunctival injections compared to topical anesthetic alone, however this was not replicated in other studies by Blaha, Kozak, Friedman or Davis [17]. 

Our subgroup analysis of treatment naïve patients showed a lower pain score for pledget group compared to topical alone group, although not statistically significant. The level of pain on VAS also subsided much quicker in this subgroup of treatment naïve patients compared to placebo. This may suggest that for naïve patients, who may be more sensitive to pain, will benefit from additional anesthesia provided through the addition of a pledget. The pain and discomfort of IVI may become more tolerable after repeated injections but naïve patients may actually benefit from the adjunctive effect of a pledget. This finding may help improve pain control, and thus future compliance, in patients receiving injections for the first time. A large sample size of treatment naïve patients may be needed to help validate these findings.

Adjuncts to the use of anesthetics have also been studied. Music has been proven to be effective in alleviating anxiety during intravitreal injections but the statistical significance of its potential analgesic effect has not been successfully proven [20, 21]. Other methods that do not necessarily relieve pain but improve overall experience during the procedure include the use of a neck pillow, presence of extra staff, having a verbal warning prior to the injection, receiving injections for both eyes on the same occasion and having positive interactions with the doctor [22, 23]. Specific strategies also appeared to be more effective for females, such a having extra staff, having their hand held or having a stress ball [22]. In our study, we had a male predominance, which may have affected pain perception and pain and anxiety alleviating factors. In the past, it has been shown that women were more anxious than men prior to IVI, and that preprocedural anxiety was associated with increased perceived pain [3]. Younger patients between 30 and 60 also tend to prefer background music during their injection as a method of anxiolytic [22]. Age and sex are thus also important factors to consider when attempting to improve the overall patient experience during IVI.

### Strengths and limitations

There are several strengths to our study. The prospective randomized design allowed us to directly compare droplet anesthesia to that of the addition of a pledget anesthesia. The crossover design allows each patient to act as their own control and helps to eliminate between-subject variability, which is crucial for a study evaluating something as subjective as pain perception. This helped to minimize bias and confounding factors that can influence pain perception.

One limitation is that there were several investigators performing the IVIs. We had a total of 5 doctors performing IVI and although trained to perform pledget anesthesia and IVI in the same manner, there may still be minute differences in technique. On the other hand, this potential variation of the injection procedure is more representative of the real-world experience. Many patients will receive injections and therefore anesthesia from more than one doctor throughout the course of their treatment. Furthermore, we only utilized VAS as the only pain evaluation method, which may not have been thorough enough to gather information on something as subjective as pain. Another potential limitation is that we included treatment naive patients in our study, which may induce recall bias. The first experience of an IVI would most likely be more pain provoking than subsequent experiences when the patients have been primed. Lastly, as the difference in pain perception may be very small, the number of patients could have been increased to increase the study power. This is particularly true for the subgroup of treatment naïve patients (*n* = 11). Further studies including a larger sample of patients may be necessary to increase robustness of the results.

## Conclusions

IVIs are one of the most commonly performed procedures in medicine today and their role continues to expand. With this, the pain associated with injections and resulting distress will grow along with it. The optimal method of analgesia to alleviate the associated pain remains unknown, but our results show that pledget provides no additional benefit to topical anesthesia. There may be potential benefit in adjunctive anesthesia treatment naïve patients, but further studies with larger sample size are needed to validate this finding.

## Electronic Supplementary Material

Below is the link to the electronic supplementary material.

Supplementary Material 1

## Data Availability

No datasets were generated or analysed during the current study.

## Abbreviations

AMD: Age related macular degeneration
DME: Diabetic macular edema
CRVO: Central retinal venous occlusion
BRVO: Branched retinal venous occlusion
HCL: Hydrochloride
IVI: Intravitreal injections
NS: Normal saline
VAS: Visual analog scale
