# Supplementary figures and images for: Comparison of different topical anesthetic methods for intravitreal injections: a randomized crossover study
Source: Int J Retina Vitreous. 2025 Mar 26;11:36. doi: 10.1186/s40942-025-00649-6 (PMC11948794; doi:10.1186/s40942-025-00649-6)

**Appendix 1**


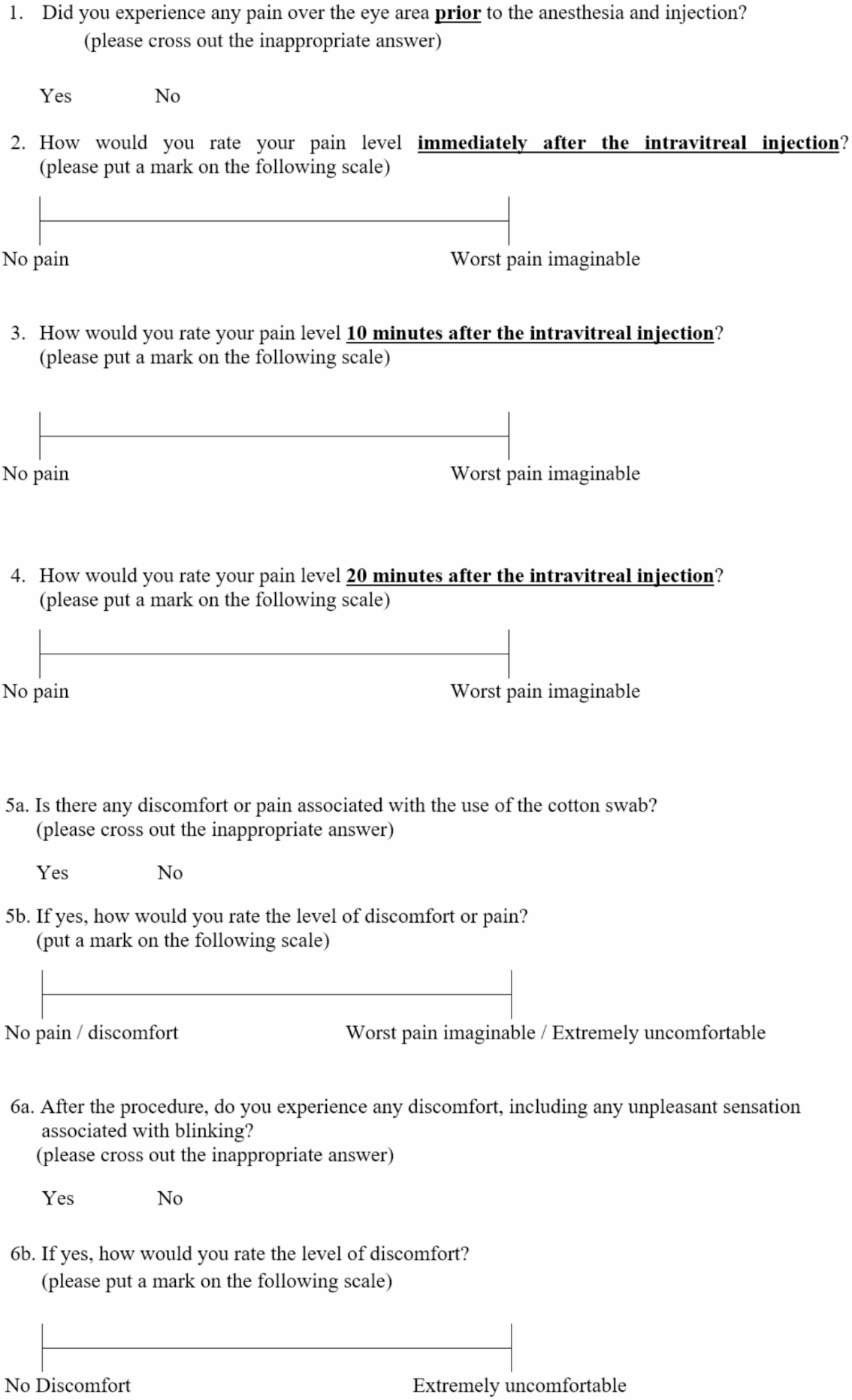

Supplement: Supplementary file 1 — Supplementary Material 1 [file 40942_2025_649_MOESM1_ESM.docx]
